# Changes of Peripheral T Cells in Systemic Lupus Erythematosus Patients

**DOI:** 10.1002/iid3.70156

**Published:** 2025-02-21

**Authors:** Juanfeng Lao, Rongjun Huang, Rongcai Wu, Yulin Yuan

**Affiliations:** ^1^ Department of Laboratory Medicine, Guangxi Academy of Medical Sciences The People's Hospital of Guangxi Zhuang Autonomous Region Nanning Guangxi China; ^2^ Department of Rheumatology and Immunology, Guangxi Academy of Medical Sciences The People's Hospital of Guangxi Zhuang Autonomous Region Nanning Guangxi China

**Keywords:** activation molecules, inhibitory molecules, lymphocyte, SLE, T cell

## Abstract

**Background:**

Efficient indicators for evaluating the imbalance of lymphocyte function were crucial to clinical therapy in systemic lupus erythematosus (SLE) patients. This study aimed to find biomarkers to assess lymphocyte‐mediated immune response in SLE patients.

**Methods:**

A total of 81 SLE patients (non‐active: *n* = 35, active: *n* = 46) and 70 healthy donors were recruited in the study. Peripheral blood was obtained, and flow cytometry was used to detect circulating lymphocytes.

**Results:**

Data showed that the counts of CD3^+^T, CD4^+^T, CD8^+^ T, and NK cells were decreased in active SLE patients compared with non‐active SLE patients and healthy donors. The counts of peripheral T cells were increased in responders but decreased in non‐responders among active patients. In addition, an increase in B cell counts was found in active SLE patients compared with those in the other two groups. Active SLE patients showed higher percentages of memory T cells but lower naive T cells than those in non‐active SLE patients and healthy controls. Activation molecules (CD38 and HLA‐DR) and inhibitory molecule PD‐1 expressions on T cells were significantly higher but percentages of CD28^+^CD8^+^T cells were lower in active SLE patients compared with those in the other two groups.

**Conclusion:**

This study indicated that monitoring the alterations of circulating lymphocyte counts and surface molecules may be helpful to assess disease activity of SLE patients, even discriminate active and non‐active patients, which was beneficial to choose the best treatment option in clinical therapy.

## Graphical abstract

Compared with non‐active SLE patients and healthy donors, the counts of CD4^+^T, CD8^+^ T, and NK cells from peripheral blood in active SLE patients were decreased, while the counts of B cells were increased. Moreover, active SLE patients showed an obvious increase in the frequencies of CD38, HLA‐DR, and PD‐1 but a decrease in the percentages of CD28 in CD4^+^ and CD8^+^T.

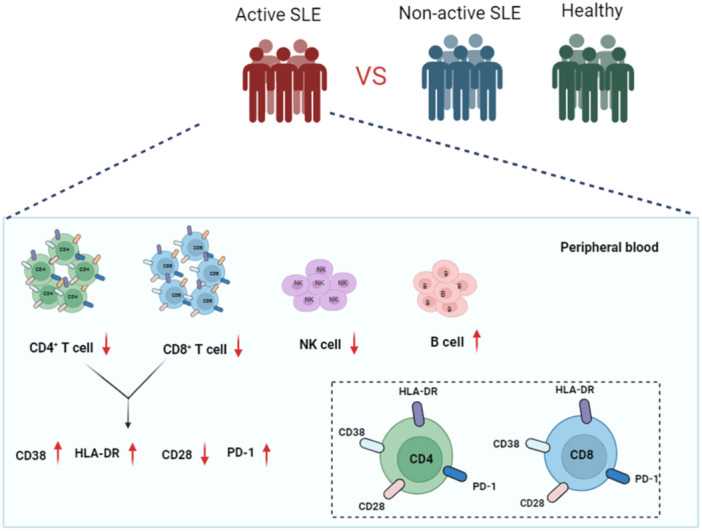

## 1 Introduction

Systemic lupus erythematosus (SLE) was an autoimmune inflammatory connective tissue disease that often affected multiple organs [1]. The typical feature of SLE was excessive inflammatory responses, which resulted in an increase in auto‐antibodies and immune complexes, ultimately leading to organ dysfunction [2]. SLE was much more common in women, with a female‐to‐male ratio of 9 to 1 [3]. The specific mechanism of SLE was not yet fully understood, but knowledge of the pathogenesis of SLE was constantly evolving.

Antinuclear antibodies (ANA) were typical diagnose markers in SLE [4]. Serum anti‐C1q, anti‐dsDNA, anti‐Nucl, and anti‐His levels were significantly higher in SLE patients and positively associated with disease activity [5]. Serum complements such as C3 and C4 were negatively related to the Systemic Lupus Erythematosus Disease Activity Index (SLEDAI) [6]. C1q deficiency was found in some lupus‐like syndrome patients [7]. Some serum proteins such as chemokines, adhesion molecules, and cytokines have been reported to be potential biomarkers of SLE [8, 9]. Circuiting levels of S100 proteins were meaningful to discriminate between SLE patients and healthy people [10]. Previous studies observed that some circulating microRNAs (miRNAs) and circular RNAs (circRNAs) were identified as biomarkers of SLE [11, 12]. It was reported that miR‐181a and miR‐203 showed diagnostic values for active SLE [13]. Recently, researchers are working to investigate the role of immune cells in SLE.

T and B‐lymphocytes played a significant role in the pathogenesis of SLE [14]. Auto‐antibodies were produced by hyperactive B cells [15]. B cell‐activating factor (BAFF), which plays a vital role in promoting self‐reactive B cell survival, has been reported to be closely associated with disease activity in SLE patients [16]. Anti‐BAFF was widely used for the treatment of SLE [17]. Killing circulating plasma cells by targeting SLAMF7 and CD38 on NK cells via enhancing its cytotoxic function may be an effective therapeutic strategy [18]. In the pathogenesis of SLE, B cell function was supported by T cells [19]. T helper cells promoted the production of auto‐antibodies by B cells via secreting multiple cytokines such as IL‐2 and IL‐17 [20, 21]. In SLE patients, CD8^+^ T cells expressed activation markers such as ICOS, CXCR5, Eomes, and T‐bet, cytotoxic and inflammatory molecules which were responsible for exacerbating the disease pathology [22, 23]. Previous study reported that SLE patients displayed lower proportions of NK cells, DC, Treg cells, and CD4^+^/CD8^+^ T‐cell ratios and higher proportions of B cells [24, 25, 26, 27]. These findings indicated that monitoring changes of peripheral blood cell subsets may be an effective way to evaluate SLE disease activity and efficacy of treatment.

In this study, immune cells including T cells, B cells, and NK cells from peripheral blood of SLE patients and immune response markers of T cells were measured by flow cytometry. Data displayed different characteristics of lymphocyte subsets in peripheral blood of active SLE patients compared to healthy donors. This study showed a decline in the counts of T lymphocyte subsets in active SLE patients compared to non‐active patients and healthy controls. Moreover, surface markers of T cells including CD38, HLA‐DR, and PD‐1 were markedly increased in active SLE patients. Therefore, monitoring peripheral T cells may be an effective way to reflect disease progression in SLE.

## 2 Materials and Methods

### 2.1 Human Donors

A cohort of 81 SLE patients who met the criteria for the classification of SLE formulated by the American College of Rheumatology revised in 1997 [28] were recruited at The People's Hospital of Guangxi Zhuang Autonomous Region from October 2022 to April 2023 (Table 1). Medications including glucocorticoid and additional immunosuppressive agents such as cyclophosphamide, cyclosporine, and azathioprine, were used to treat active SLE patients based on their condition. Hydroxychloroquine were used to treat the non‐active SLE patients, which can reduce the frequency of disease flares (particularly of lupus nephritis), contribute to the maintenance of remission, prolong the onset of disease, and reduce the risk of complications. According to the SLEDAI 2002 [6], patients were divided into different activity groups: non‐active (SLEDAI ≤ 4) group and active (SLEDAI ≥ 5) group. This study recruited 70 healthy volunteers without SLE and other autoimmune disease from the Health Examination Center of The People's Hospital of Guangxi Zhuang Autonomous Region between September 2022 to March 2023 (Table 1). The control groups were age‐ and sex‐matched.

**Table 1:** Demographic and clinical variables of non‐active SLE and active SLE patients.

	Healthy donors	Non‐active SLE	Active SLE
	*n* = 70	*n* = 35	*n* = 46
Mean age (years)	37.60 ± 8.12	37.17 ± 14.77	34.98 ± 14.92
Sex			
Femalen, *n* (%)	63 (90.00)	31 (88.57)	42 (91.30)
SLEDAI	0	≤ 4	≥ 5
ANA (positive), *n* (%)	0 (0)	27 (77.14)	45 (97.82)
Anti‐dsDNA, IU/mL	< 1.00	4.86 ± 11.08	20.17 ± 27.27
Complement 3, g/L	0.93 ± 0.16	0.65 ± 0.29	0.45 ± 0.22
Complement 4, g/L	0.23 ± 0.07	0.25 ± 0.26	0.09 ± 0.06
PLT (×10^9^/L)	276.22 ± 55.13	235.54 ± 94.62	210.85 ± 108.89
WBC (×10^9^/L)	6.28 ± 1.28	5.65 ± 2.48	6.01 ± 3.92
RBC (×10^12^/L)	4.94 ± 0.76	4.16 ± 0.87	3.77 ± 0.75
Hb (g/L)	133.56 ± 16.23	118.49 ± 14.27	103.24 ± 20.23
Clinical features			
CNS, *n* (%)	0 (0.0)	0 (0.0)	13 (28.3)
Vasculitis, *n* (%)	0 (0.0)	0 (0.0)	5 (10.9)
Musculoskeletal, *n* (%)	0 (0.0)	0 (0.0)	10 (21.7)
Renal, *n* (%)	0 (0.0)	6 (17.1)	36 (76.3)
Mucocutaneous, *n* (%)	0 (0.0)	0 (0.0)	22 (47.8)
Serositis, *n* (%)	0 (0.0)	0 (0.0)	2 (4.3)
Hematological, *n* (%)	0 (0.0)	2 (5.7)	18 (39.1)

*Note:* Data were shown by mean ± SD.

### 2.2 Flow Cytometry Assay

Peripheral blood samples were collected and stained for BD Multitest 6‐color TBNK reagent (No.662967, BD) in BD Trucount tubes, CD3 (Clone SK7, BD), CD4 (Clone SK3, BD), CD8 (Clone SK1, BD), CD45RA (Clone L48, BD), CD62L (Clone SK11, BD), CD28 (Clone L293, BD), CD38 (Clone HB7, BD), HLA‐DR (Clone L243, BD), PD‐1 (Clone MIH4, BD) in normal test tubes that without beads for 15 min at room temperature. Then add 450 µL RBC Lysis Buffer to BD Trucount tubes and 1 mL to normal test tubes. After 10 min, samples in BD Trucount tubes were ready to be detected. Samples in normal test tubes should be centrifuged and washed three times with FACS buffer. Finally, add 300 µL FACS buffer to the sample tubes for detection.

### 2.3 Statistical Analysis

In the study, data were analyzed by GraphPad Prism 8.0 Software (San Diego, CA). Mann−Whitney test was used for nonparametric tests. Data were shown as mean ± SEM unless indicated otherwise. *p* < 0.05 was considered statistically significant.

## 3 Results

### 3.1 Clinical Characteristics

Clinical characteristics of participants were shown in Table 1 and Table S1. A total of 81 SLE patients and 70 healthy donors were included in this study (Table 1). The mean ages of these patients were 36 years old, of which 90% were female. On admission, 35 and 46 patients met the diagnostic criteria for non‐active and active disease, respectively. Most active patients showed typical clinical features of SLE.

### 3.2 Circulating Lymphocytes Counts Were Decreased in Active SLE Patients

**Figure 1:**
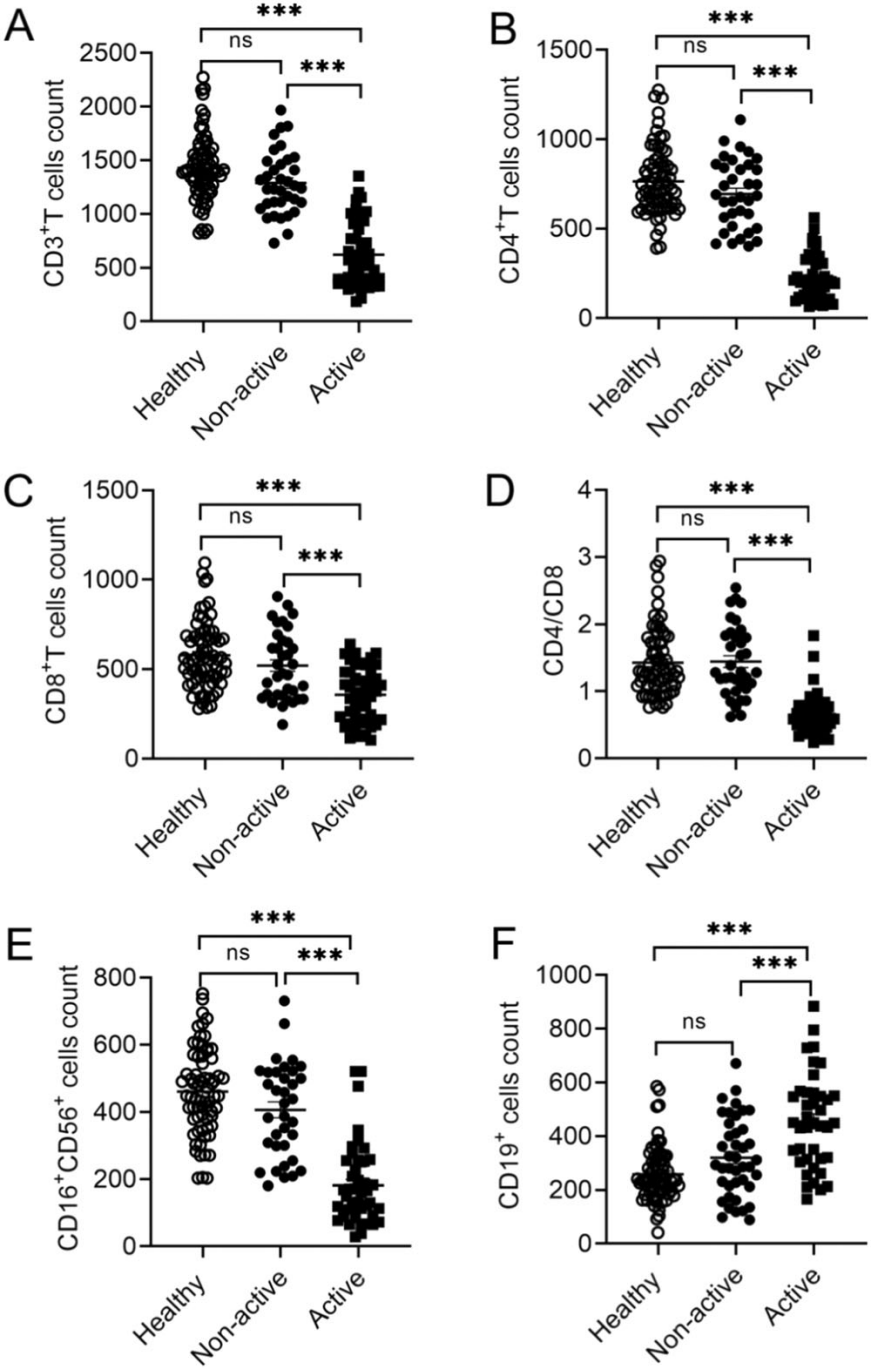
Comparison of immune cell populations counts in healthy donors and SLE patients. Cell subsets were gated in total peripheral blood from healthy donors (*n* = 70), non‐active (*n* = 35), and active SLE patients (*n* = 46) and absolute counts and CD4/CD8 were compared in the groups: (A) CD3^+^ T cells, (B) CD4^+^ T cells, (C) CD8^+^ T cells, (D) CD4/CD8, (E) CD19^+^ B cells, (F) CD3^−^CD16^+^CD56^+^ NK cells. Data were presented as means ± SEM. ns, not significant, ***p < 0.001.

**Figure 2:**
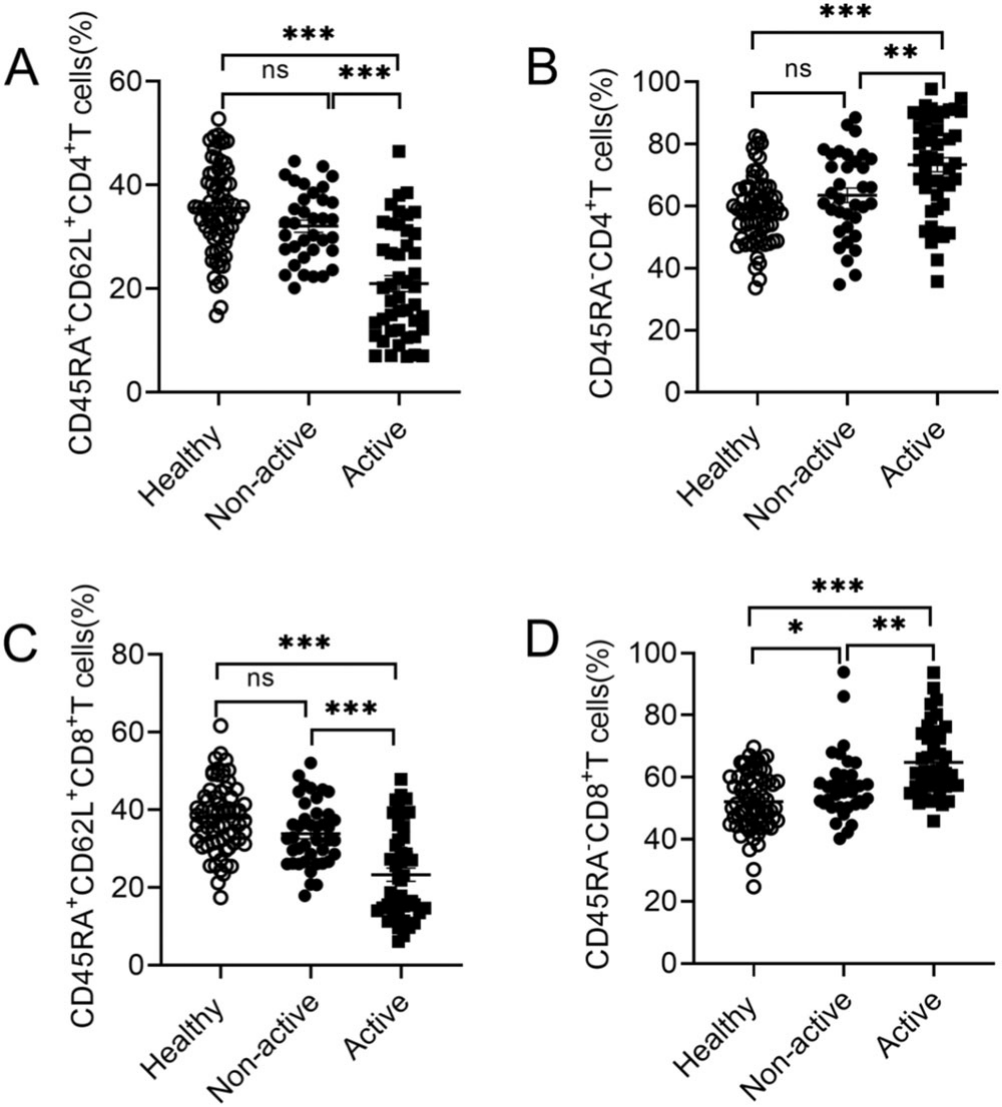
Comparison of naive and memory T cell populations in healthy donors and SLE patients. Cell subsets were gated in total peripheral blood from healthy donors (*n* = 70), non‐active (*n* = 35), and active SLE patients (*n* = 46), and percentages of naive (CD45RA^+^CD62L^+^) and memory (CD45RA^−^) T cells were compared in the groups: (A) CD45RA^+^CD62L^+^CD4^+^T cells, (B) CD45RA^−^CD4^+^T cells, (C) CD45RA^+^CD62L^+^CD8^+^T cells, (D) CD45RA^−^CD8^+^T cells. Data were presented as means ± SEM. ns, not significant, *p < 0.05,**p < 0.01,***p < 0.001.

**Figure 3:**
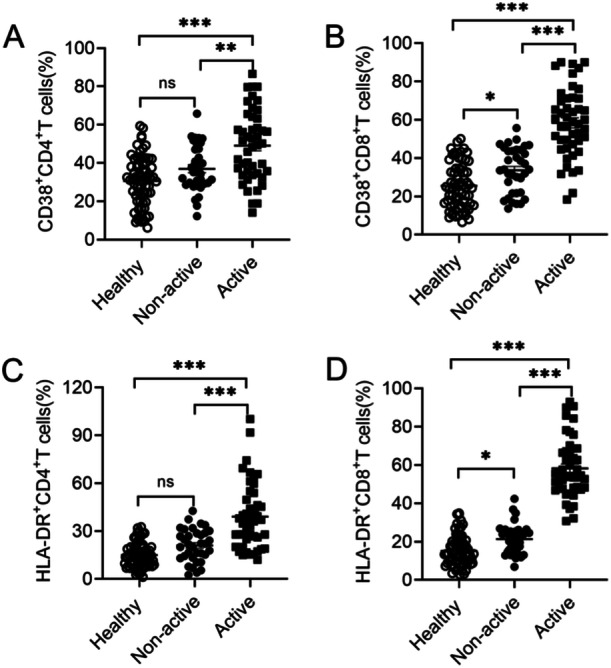
Activation molecules expression on T cells from PBMC in healthy donors and SLE patients. Frequencies of CD38^+^ (A, B) and HLA‐DR (C, D) cells gated on total CD4^+^ or CD8^+^T cells from PBMC in healthy donors (*n* = 70), non‐active (*n* = 35), and active SLE patients (*n* = 46). Data were presented as means ± SEM. ns, not significant,*p < 0.05,**p < 0.01,***p < 0.001.

To understand the changes of circulating immune response, we detected peripheral blood cell subsets from SLE patients. Peripheral blood samples were collected and lymphocytes were analyzed by flow cytometry (Supporting Information S2: Figure 1 and Figure 1). Results showed that circulating total T lymphocyte (CD3^+^cells) counts were decreased in active SLE patients compared with those from healthy control and non‐active SLE patients (Figure 1A). Active SLE patients showed abnormal immune function, including fewer CD4^+^T cells, CD8^+^T cells, and CD16^+^CD56^+^NK cell counts (Figure 1B,C,E). The ratio of CD4^+^T and CD8^+^T was significantly decreased in the active group as compared with healthy control and the non‐active group (Figure 1D), which indicated that the immune system was imbalanced in active SLE patients. Furthermore, CD19^+^B cell counts were increased in active SLE patients (Figure 1F), which may be associated with abnormal auto‐antibody secretion. There was a negative correlation between CD3^+^T, CD4^+^T, CD8^+^T, and NK cells with CD19^+^B cells in active SLE patients (Supporting Information S2: Figure 2A−D). A positive correlation between CD19^+^B cells with anti‐dsDNA was found (Supporting Information S2: Figure 3A). Data showed a negative between CD4^+^T and CD8^+^T cells with anti‐dsDNA levels in active SLE patients (Supporting Information S2: Figure 3B,C). These results indicated that changes of peripheral lymphocyte counts were closely associated with patients' disease activity.

### 3.3 Changes of Naive and Memory T Cells Populations in Active SLE Patients

**Figure 4:**
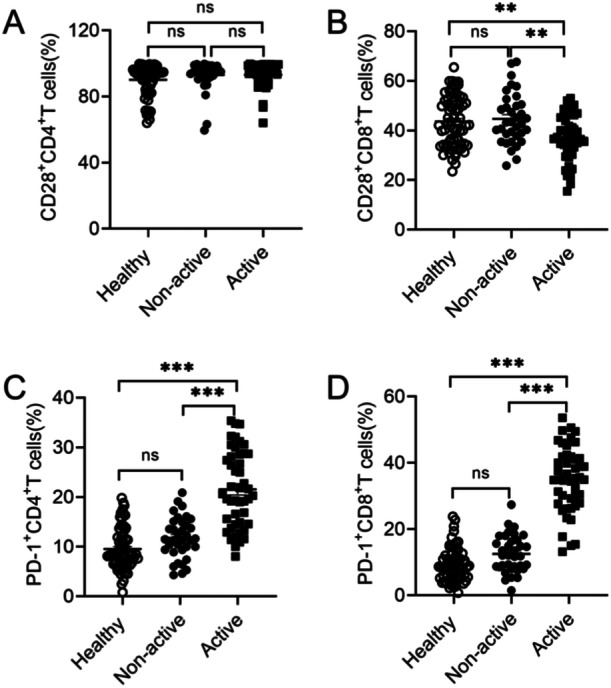
Expression of co‐stimulatory molecule CD28 and co‐inhibitory molecule PD‐1 on T cells from PBMC in healthy donors and SLE patients. Frequencies of CD28^+^CD4^+^T cells (A), CD28^+^CD8^+^T cells (B), PD‐1^+^CD4^+^T cells (C), and PD‐1^+^CD8^+^T cells (D) from PBMC in healthy donors (*n* = 70), non‐active (*n* = 35), and active SLE patients (*n* = 46). Data were presented as means ± SEM. **p < 0.01,***p < 0.001.

We next detected naive and memory T cell populations by flow cytometry (Supporting Information S2: Figure 4 and Figure 2). Data showed that active SLE patients displayed lower percentages of naive CD4^+^T cells (CD45RA^+^CD62L^+^CD4^+^T cells) compared to healthy donors and non‐active SLE patients (Figure 2A). On the contrary, the percentages of memory CD4^+^T cells (CD45RA^−^CD4^+^T cells) in active SLE patients were higher than those of healthy donors and non‐active SLE patients (Figure 2B). There was no difference in naive and memory CD4^+^T cells between healthy donors and non‐active SLE patients (Figure 2A,B). Similarly, active SLE patients showed decreased frequencies of naive CD8^+^T cells (CD45RA^+^CD62L^+^CD8^+^T cells) and increased frequencies of memory CD8^+^T cells (CD45RA^−^CD8^+^T cells) (Figure 2C,D). There was no significant difference in naive CD8^+^T cells between healthy donors and non‐active SLE patients (Figure 2C). Non‐active SLE patients displayed higher percentages of memory CD8^+^T cells than healthy donors (Figure 2D). The differences in the percentages of naive and memory T cells between SLE patients and healthy donors indicated that active SLE patients displayed imbalanced immune cell subsets.

### 3.4 Expression of Activation Molecules on T Cells Were Increased in Active SLE Patients

To better understand T cell responses in SLE, we detected activation molecule expression on peripheral blood T cells from the patients. Results demonstrated that active SLE patients showed higher expression levels of CD38 on both CD4^+^T and CD8^+^T cells than healthy controls as well as non‐active SLE patients (Figure 3A,B). Moreover, active SLE patients displayed higher levels of HLA‐DR on both CD4^+^T and CD8^+^T cells compared with healthy controls and non‐active SLE patients (Figure 3C,D). Expression levels of CD38 and HLA‐DR on CD4^+^T cells were no difference between healthy controls and non‐active SLE patients (Figure 3A,C). However, non‐active SLE patients showed higher expression levels of these activation markers in CD8^+^T cells compared to healthy controls (Figure 3B,D). These results indicated that CD8^+^T cells were also activated in non‐active SLE patients. There was a negative correlation between CD38^+^CD4^+^T, CD38^+^CD8^+^T, HLA‐DR^+^CD4^+^T, and HLA‐DR^+^CD8^+^T cells with anti‐dsDNA levels in active SLE patients (Supporting Information S2: Figure 3D−G). The increase of CD38 and HLA‐DR on peripheral blood T cells from active SLE patients indicated that T cells immune responses were abnormally activated in active patients.

### 3.5 Expression of Co‐Stimulatory Molecule CD28 and Co‐Inhibitory Molecule PD‐1 on T Cells in Active SLE Patients

To further understand patients' responses, we next monitored the alteration of co‐stimulatory molecule CD28 and co‐inhibitory molecule PD‐1 on T cells from SLE patients. Results demonstrated that percentages of CD28 were no dereference on CD4^+^T cells between healthy donors, active and non‐active SLE patients (Figure 4A). However, active SLE patients showed lower expression levels of CD28 on CD8^+^T cells compared with the other two groups (Figure 4B). Results also demonstrated that active SLE patients displayed an increase in expression of PD‐1 on CD4^+^T cells (Figure 4C). Similarly, results were also observed on CD8^+^T cells (Figure 4D). There was no difference in expression of PD‐1 on both CD4^+^ and CD8^+^ T cells between healthy donors and non‐active SLE patients (Figure 4C,D). The different changes of PD‐1 expression between healthy donors, active and non‐active SLE patients may suggest that T‐cell exhaustion occurred during disease progression. Co‐stimulatory molecule CD28 and co‐inhibitory molecule PD‐1 may be vital biomarkers for immune assessment in SLE patients.

### 3.6 Changes of T Cell Counts in Responsive and Nonresponsive Active SLE Patients

**Figure 5:**
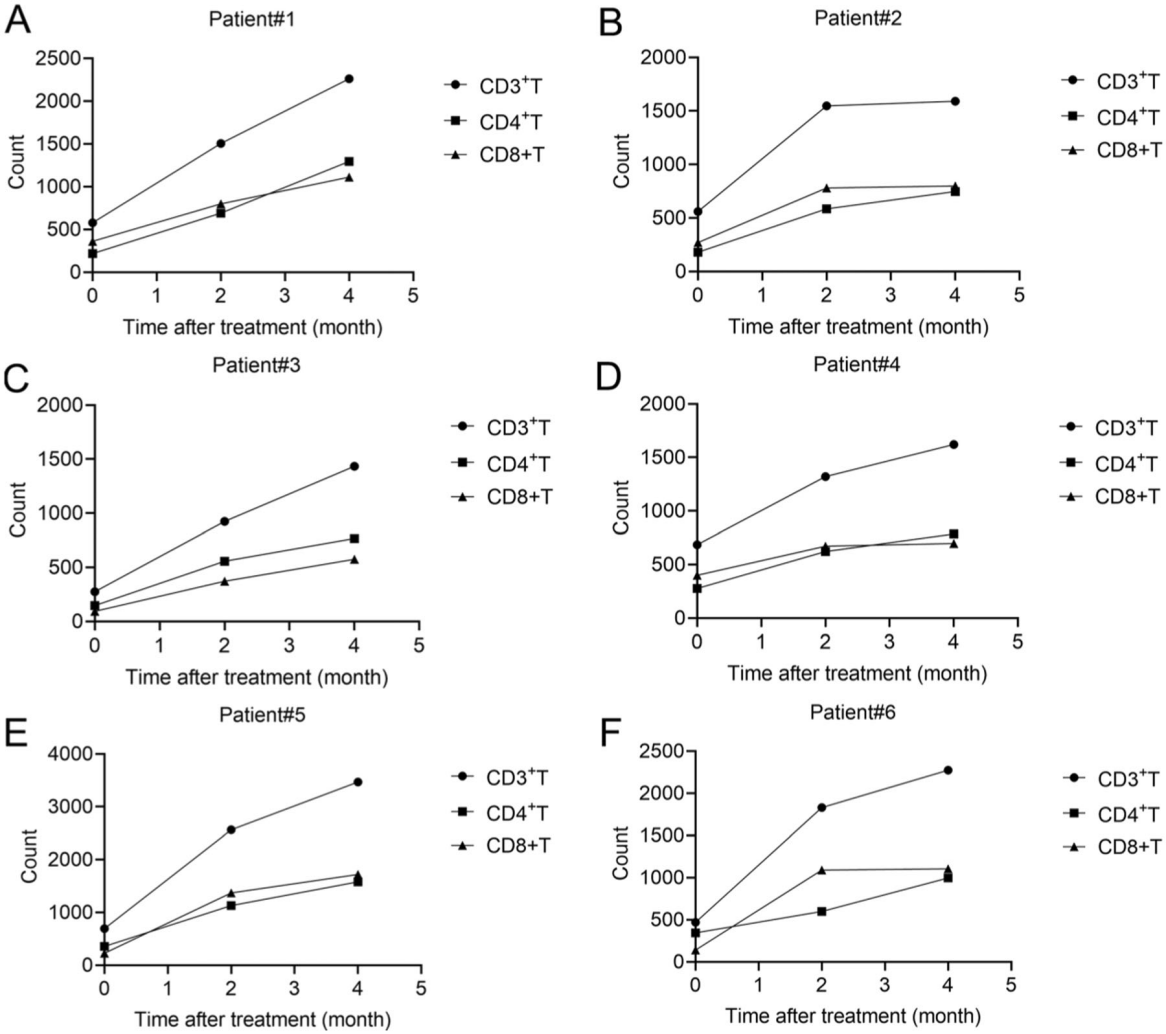
Changes of CD3^+^T, CD4^+^T, and CD8^+^T cell counts in six SLE patients (A−F) with effective treatment at the indicated time. (A) Patient#1, (B) Patient#2, (C) Patient#3, (D) Patient#4, (E) Patient#5, and (F) Patient#6.

**Figure 6:**
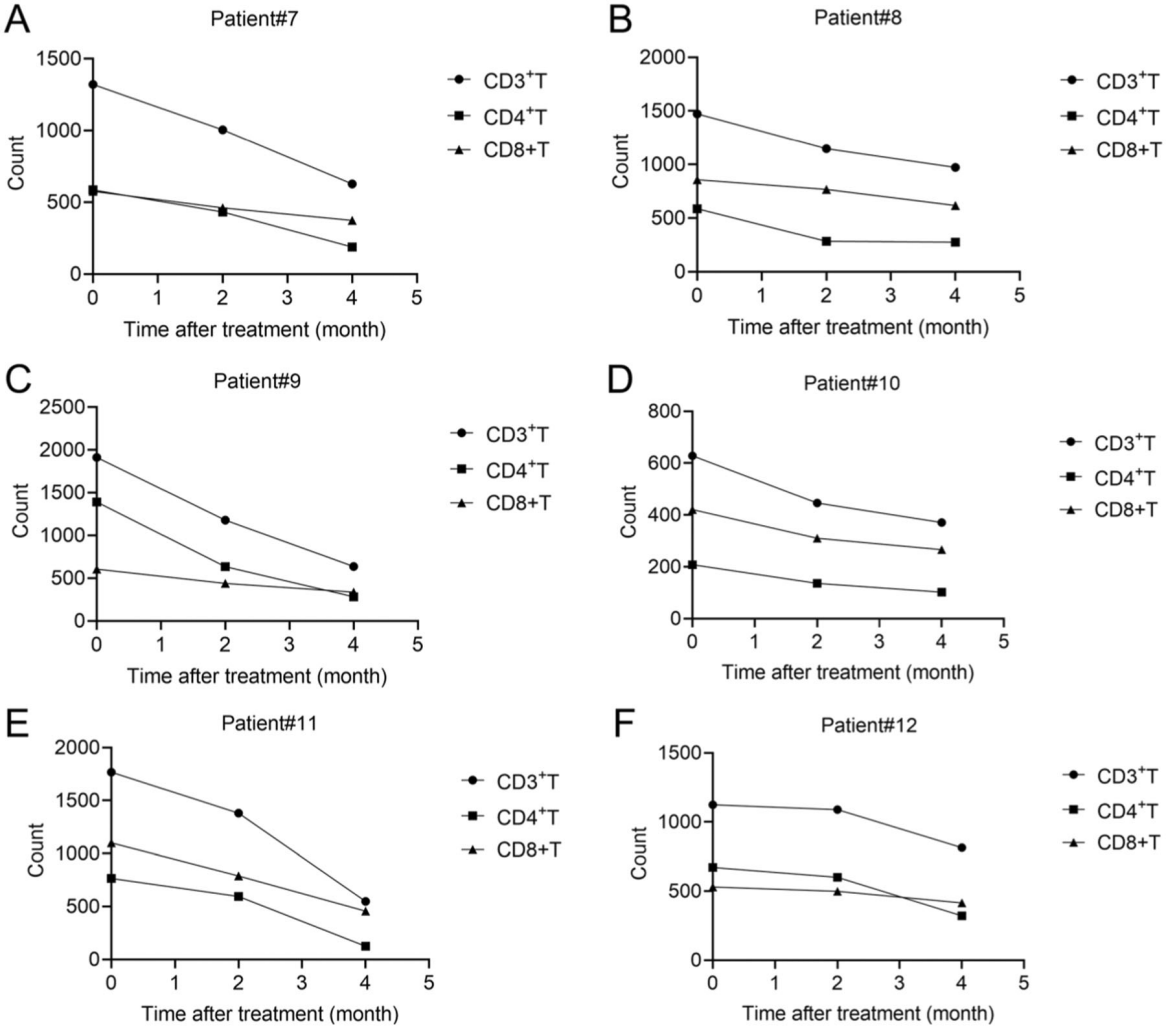
Changes of CD3^+^T, CD4^+^T, and CD8^+^T cell counts in six SLE patients (A−F) with ineffective treatment at the indicated time. (A) Patient#7, (B) Patient#8, (C) Patient#9, (D) Patient#10, (E) Patient#11, and (F) Patient#12.

The counts of CD3^+^T, CD4^+^T, and CD8^+^T cells in active SLE patients before and after glucocorticoid treatment were analyzed. Twelve active patients were divided into two groups according to the clinical outcomes. Patient 1 to patient 6 responded to treatment and patient 7 to patient 12 did not respond to treatment (Figures 5 and 6). Data showed that the counts of CD3^+^T, CD4^+^T, and CD8^+^T cells in these cases were all at low levels before treatment (Figures 5 and 6). After treatment for 2 and 4 month, CD3^+^T, CD4^+^T, and CD8^+^T cell counts were increased in responsive patients (Figure 5A−F). However, these cells were decreased in nonresponsive patients (Figure 6A−F). These results indicated that the increased of counts of T cells in active SLE patients were associated with better outcomes.

## 4 Disscusion

SLE was a refractory autoimmune disease which affected multiple organs [6]. Monitoring the activity of disease biomarkers and immune function was an important factor in clinical management of SLE [29]. ANAs and anti‐ds‐DNA were common diagnostic parameters for SLE, while C3 and C4 levels, anti‐Sm/RNP, and anti‐cardiolipin antibodies were important disease activity biomarkers [29, 30]. Recently, monitoring the changes of peripheral blood lymphocyte counts and phenotype has been a usual approach for determining the immune status of SLE patients. This study observed that alterations in peripheral blood lymphocytes were closely associated with disease activity and treatment responsiveness of patients in SLE.

Lymphocytopenia was a common clinical manifestation in SLE patients [31]. It was confirmed that lymphocytes could be attacked by auto‐antibodies in SLE patients [32, 33]. Reduction of circulating lymphocyte subsets had been observed in SLE patients, which was correlated with disease activity [34]. Consistent with previous research, we found that peripheral blood lymphocyte counts were seriously imbalanced in active SLE patients but not non‐active patients. The lymphocyte disorder may be related to the compromised immune system of active patients.

We showed that counts of CD3^+^T, CD4^+^T, CD8^+^T, and NK cells were significantly decreased, but CD19^+^B cells were upregulated in active SLE patients compared with non‐active patients and healthy people. The increase of CD19^+^B cells may be associated with high production of auto‐antibodies in active SLE patients. Other biomarkers should be used to label activated B cells to confirm the conjecture. Naïve T cells were a cluster of inactive cells. Once encountering self‐antigen, they began to proliferate and rapidly differentiate into effector T cells [19]. After the pathogen was cleared, most effector cells died, while a few developed and became memory cells [19, 35]. Here, we observed that naïve and memory T cell subsets proportions were different in healthy people and active SLE patients. Active SLE patients showed a higher percent of memory T cells and a lower percent of naïve T cells than non‐active patients and healthy people. The reason for this change may be that the immune system of active SLE patients were over‐activated, then naïve T cells differentiated into other T cell types such as memory T cells.

Activation and inhibitory molecules on T cells were important for immune response in SLE patients. It was reported that activated naïve (aNAV) B cells of SLE patients showed a costimulatory phenotype and upregulated T‐cell costimulatory molecules after BCR and TLR‐7/TLR‐8 stimulation which led to T‐cell activation and polarization toward effector Th2 and Th17 cells [36]. aNAV B cells can present antigens and deliver costimulatory signals to T cells by upregulating CD40, CD86, IL‐21R, and HLA‐DR costimulatory molecules [36]. Once activated, T cells produced inflammatory cytokines which promoted the development of SLE disease. CD38 functioned as an activation receptor on T cells [37]. It was reported that expression of CD38 was increased on CD4^+^ and CD8^+^ memory T cells in SLE patients compared to healthy donors [38]. In this study, we also showed increased expression of CD38 on both CD4^+^ and CD8^+^T cells in active SLE patients compared with non‐active patients and healthy controls. High expression of CD38 had been reported to be associated with defective cytotoxic activity of CD8^+^T cells in SLE patients with infections [39]. We discovered that another T cell activation molecule, HLA‐DR, was also highly expressed in active SLE patients. A previous study observed that CD8^+^HLA‐DR^+^T cells showed exhausted and suppressive effects in cirrhosis, which were related to poor outcomes [40]. Here, we supposed that CD8^+^HLA‐DR^+^T cells may suppress anti‐infective immune response in SLE. There was no obvious difference in expression of CD28 on CD4^+^T cells between SLE patients and healthy donors in our study. However, the frequency of CD28^+^CD8^+^T cells was found to be increased in active SLE patients. CD28^+^CD8^+^T cells were designated as antigen‐primed cytotoxic T cells [41, 42]. CD28^+^CD8^+^T cells in active SLE patients may display defective cytotoxicity. The reason why expression of CD28 on CD8^+^T cells was decreased needed to be investigated. Except for activation molecules, expression of inhibitory molecule PD‐1 also varied in active SLE patients. We observed that PD‐1 expression on both CD4^+^ T and CD8^+^ T cells were higher in active SLE patients compared with healthy controls and non‐active patients, indicating that T cell exhaustion were observed in active patients. The causes of the imbalance of activation and inhibitory molecules on T cells might be that the immune system was over‐activated in SLE patients. Our study supposed that targeting these surface receptors maybe potential treatment options for SLE.

A previous study reported that proportions of some lymphocyte subsets in active SLE patients changed before and after treatment [43]. Here, we observed that counts of peripheral blood CD3^+^ T, CD4^+^T, and CD8^+^T cells were increased in active SLE patients responding to treatment, but decreased in patients who failed to respond to treatment. Our finding indicated that peripheral blood T cell subsets may be useful biomarkers for efficacy monitoring in SLE. However, There were some limitations in the study. First, a larger sample of patients should be included to confirm the conjecture. In addition, the correlations between the T cell subsets or surface markers and disease activity should be analyzed. Moreover, the exact mechanisms by which lymphocyte subsets regulate the disease pathology in SLE were needed further study.

## 5 Conclusion

In summary, we observed a wide range of anomalies in the counts of peripheral blood lymphocyte subsets and T cell phenotype in active SLE. Monitoring the alterations of circulating lymphocytes may be a promising way to assess the disease activity of SLE patients. Further in‐depth study should be carried out to investigate the role of T cell phenotypic changes for disease activity and efficacy monitoring in SLE.

## Author Contributions

Juanfeng Lao and Yulin Yuan initiated and designed the research. Juanfeng Lao completed the manuscript. Rongjun Huang and Rongcai Wu carried out the experiments and analyzed and/or interpreted the results. Rongjun Huang and Yulin Yuan were responsible for patient collection and analysis and contributed to the discussion of results. All authors read the final version of the manuscript and agreed to submit it for publication.

## Ethics Statement

This study was supported by the ethics committee of The People's Hospital of Guangxi Zhuang Autonomous Region (Ethics No. KY‐KJT‐2023‐128). All participants wrote informed written consent for their participation in the study.

## Conflicts of Interest

The authors declare no conflicts of interest.

## Supporting information

Supporting information.

## Data Availability

The data that support the findings of this study are available from the corresponding author upon reasonable request.
