# Supplementary material for: Changes of Peripheral T Cells in Systemic Lupus Erythematosus Patients
Source: Immun Inflamm Dis. 2025 Feb 21;13(2):e70156. doi: 10.1002/iid3.70156 (PMC11843224; doi:10.1002/iid3.70156)
Supplement: Supplementary file 1 — Supporting information. [file IID3-13-e70156-s001.docx]

**Supplementary Figures**


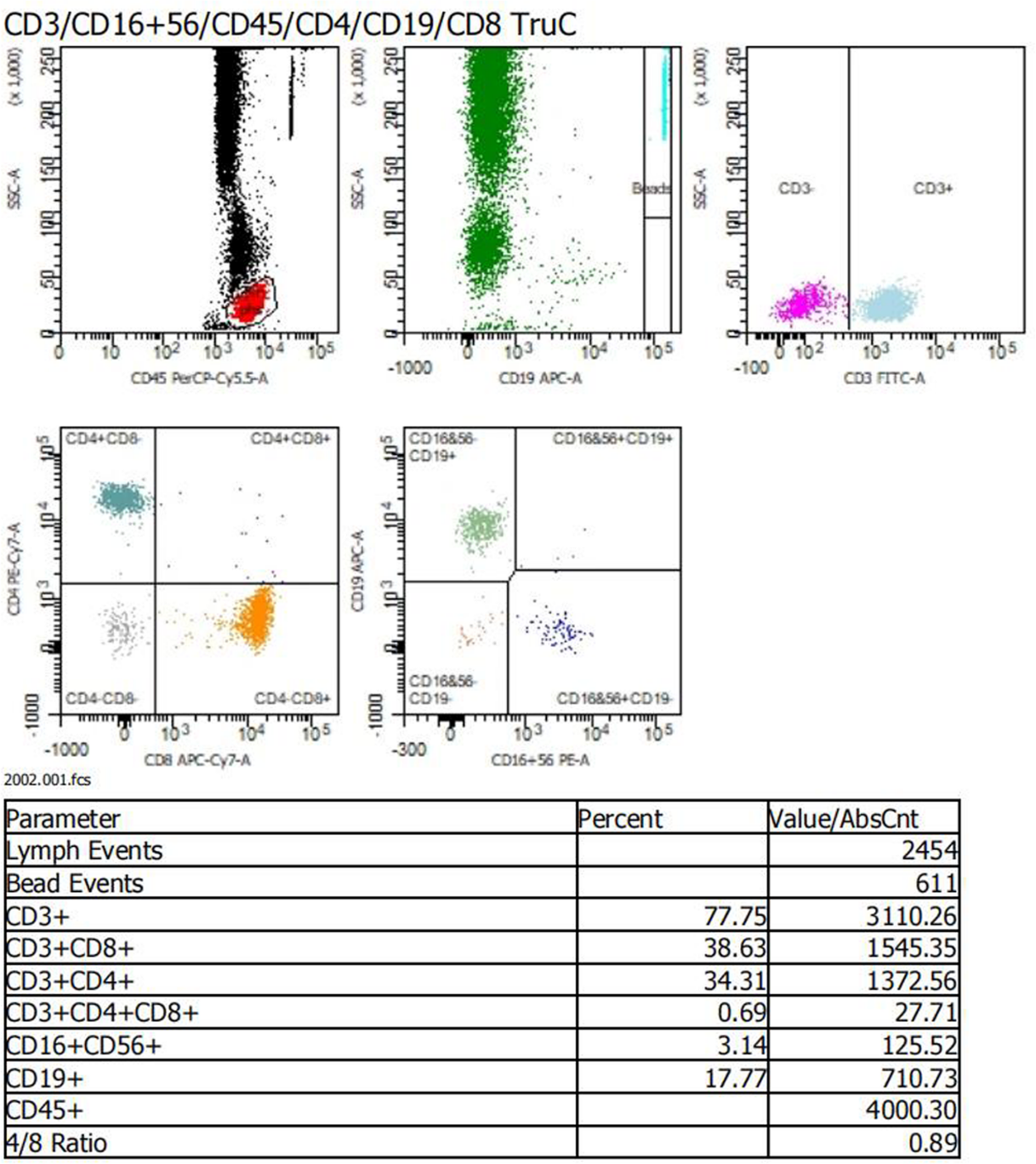


**Supplementary Figure 1** Gating strategy for detecting T, B and NK cells subsets.


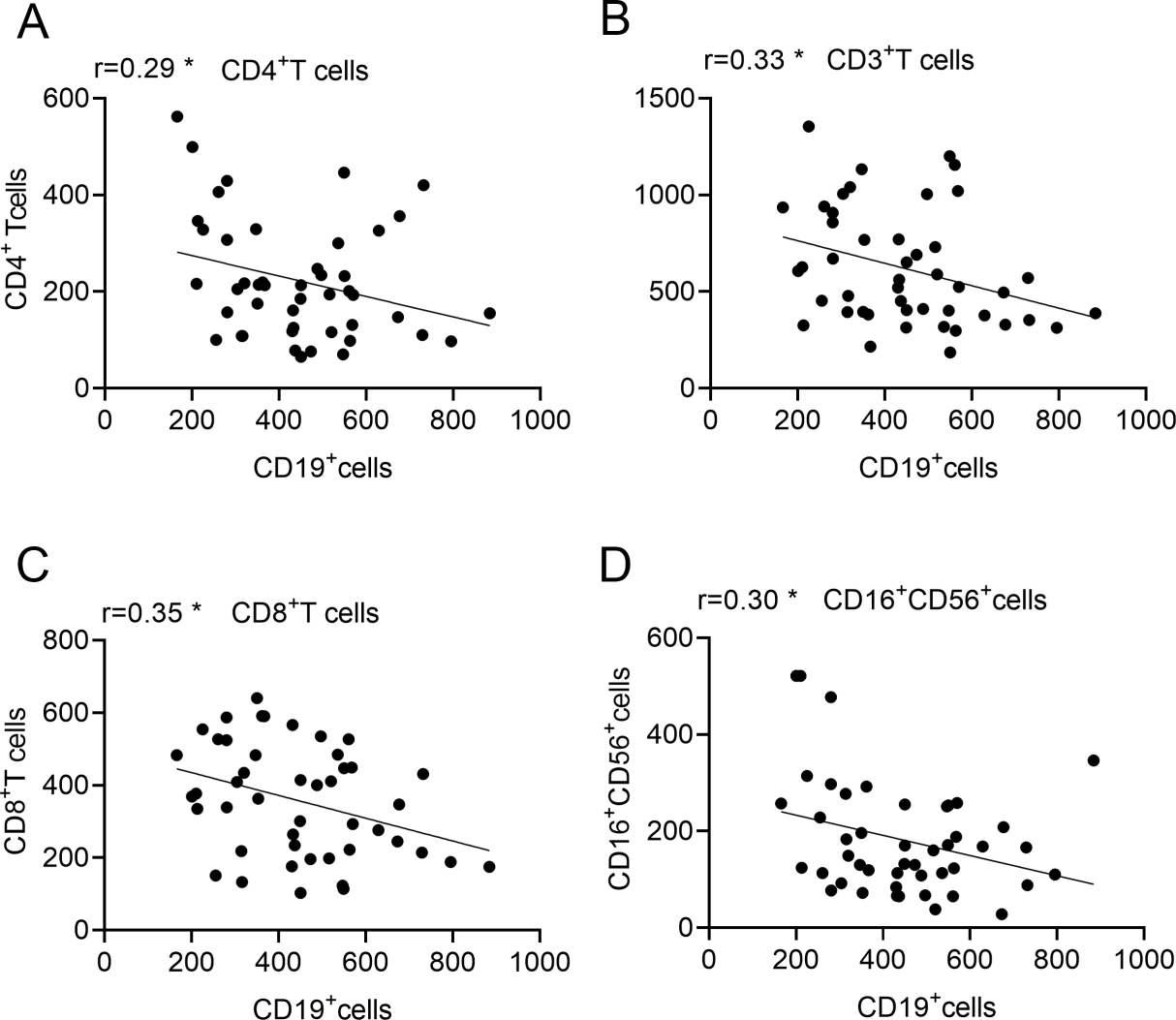


**Supplementary Figure 2** Correlation between CD3^+^T(**A**), CD4^+^T(**B**), CD8^+^T(**C**) and NK cells (**D**) with CD19^+^B cells in active SLE patients.


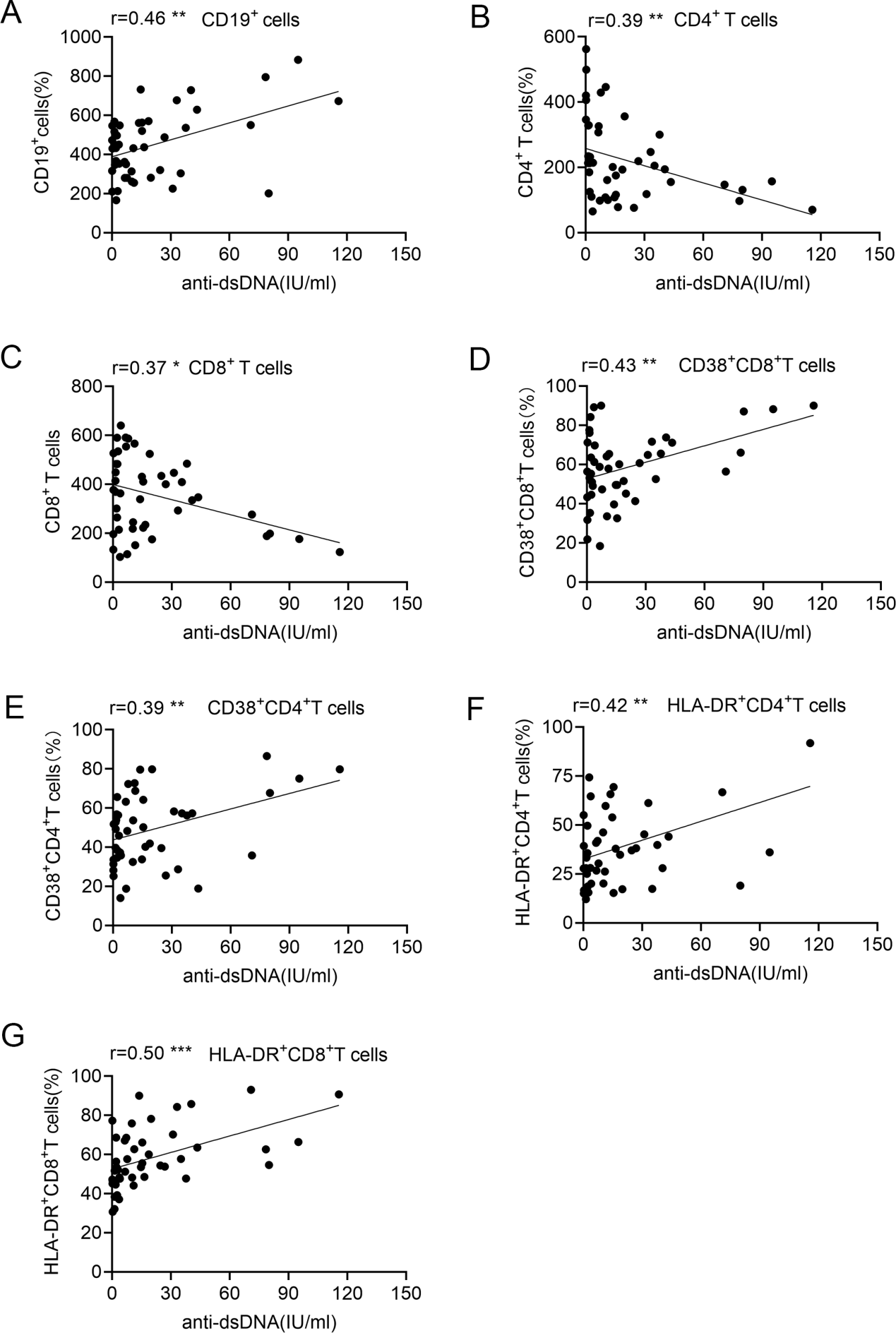


**Supplementary Figure 3** Correlation between CD19^+^B(**A**), CD4^+^T(**B**), CD8^+^T(**C**), CD38^+^CD4^+^T(**D**), CD38^+^CD8^+^T(**E**), HLA-DR^+^CD4^+^T(**F**) and HLA-DR^+^CD8^+^T(**G**), cells with anti-dsDNA levels in active SLE patients.


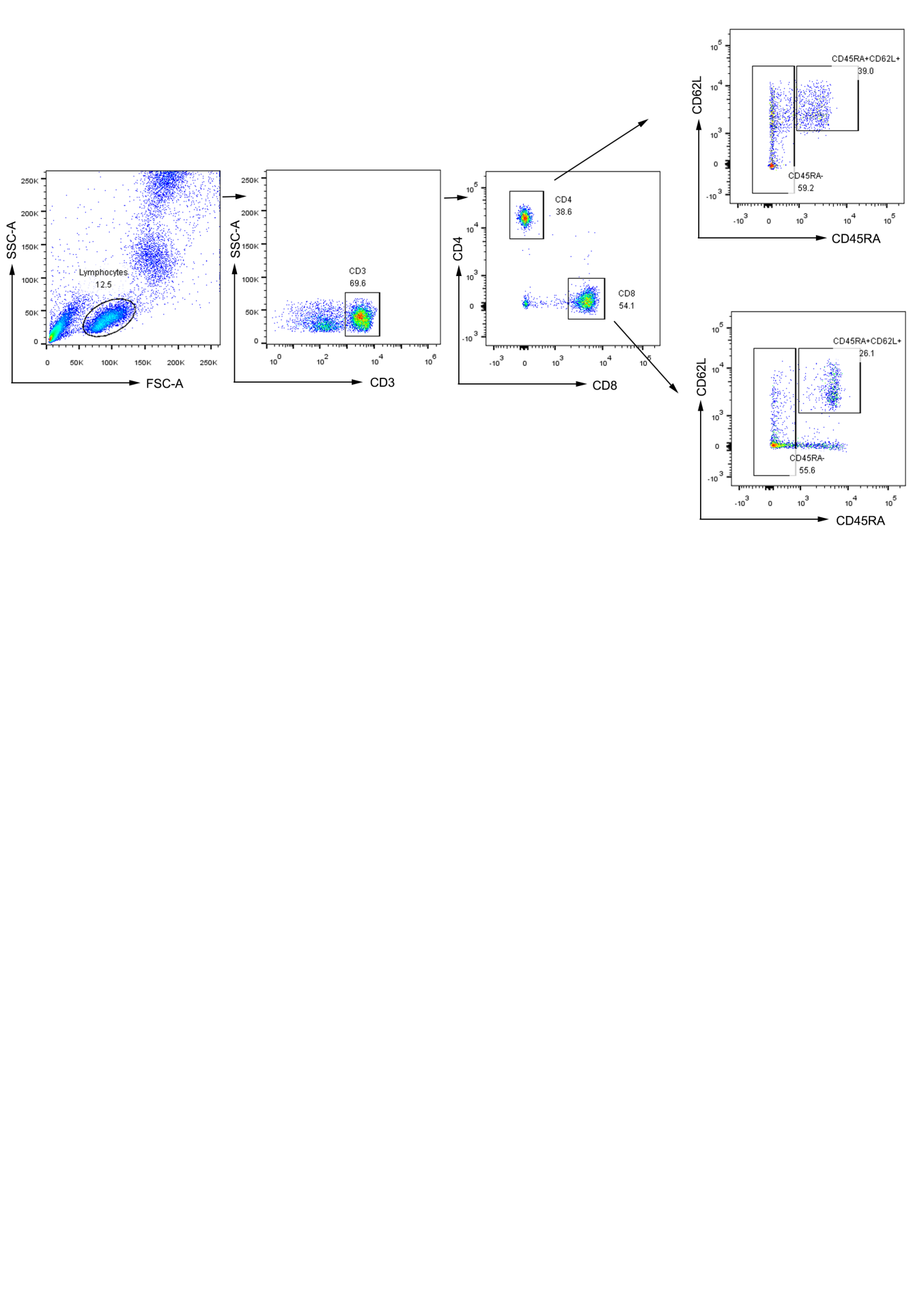


**Supplementary Figure 4** Gating strategy for detecting naive (CD45RA^+^CD62L^+^) and memory (CD45RA^-^) T cells.

| **Supplementary table 1** | | | | | | | | | | | |
| --- | --- | --- | --- | --- | --- | --- | --- | --- | --- | --- | --- |
| **Non-active patients** | | | | | | | | | | | |
| **No** | **Sex** | **Age** | **SLEDAI** | **Anti-dsDNA** | **ANA** | **C3** | **C4** | **Hb** | **PLT** | **WBC** | **RBC** |
| 1 | Woman | 34 | 0 | 0.19 | + | 1.04 | 0.26 | 107 | 347 | 8.47 | 5.21 |
| 2 | Woman | 45 | 0 | 0.35 | + | 0.04 | 0.16 | 129 | 192 | 5.61 | 4.31 |
| 3 | Woman | 63 | 0 | 0.13 | + | 0.82 | 0.16 | 118 | 294 | 8.27 | 4.08 |
| 4 | Woman | 31 | 1 | 0.4 | + | 0.75 | 0.17 | 84 | 437 | 2.76 | 3.43 |
| 5 | Woman | 60 | 1 | 4.3 | + | 0.23 | 0.31 | 111 | 31 | 6.07 | 3.85 |
| 6 | Woman | 24 | 1 | 0.08 | - | 0.66 | 0.12 | 124 | 166 | 3.79 | 4.33 |
| 7 | Woman | 46 | 1 | 0.36 | + | 1.02 | 0.25 | 128 | 245 | 5.81 | 4.41 |
| 8 | Woman | 18 | 1 | 0.32 | + | 0.98 | 0.72 | 129 | 318 | 6.35 | 3.46 |
| 9 | Woman | 49 | 1 | 0.1 | + | 0.63 | 0.87 | 72 | 444 | 14.53 | 4.71 |
| 10 | Woman | 38 | 2 | 1.01 | + | 0.48 | 0.92 | 93 | 203 | 4.67 | 2.99 |
| 11 | Man | 59 | 2 | 0.04 | - | 1.32 | 0.33 | 112 | 145 | 2.94 | 3.93 |
| 12 | Woman | 53 | 2 | 0.83 | - | 0.54 | 0.14 | 118 | 133 | 6.52 | 3.97 |
| 13 | Woman | 63 | 2 | 2.92 | + | 0.64 | 0.05 | 120 | 269 | 6 | 5.67 |
| 14 | Woman | 34 | 2 | 0.15 | + | 0.72 | 0.18 | 127 | 162 | 3.2 | 2.19 |
| 15 | Man | 24 | 2 | 0.35 | + | 0.63 | 0.21 | 132 | 225 | 5.93 | 4.77 |
| 16 | Woman | 32 | 2 | 1.43 | - | 0.78 | 0.31 | 118 | 223 | 2.59 | 2.12 |
| 17 | Woman | 26 | 3 | 0.98 | - | 0.98 | 0.15 | 125 | 161 | 5.26 | 2.56 |
| 18 | Woman | 49 | 3 | 0.36 | + | 0.7 | 0.21 | 138 | 222 | 6.01 | 4.47 |
| 19 | Woman | 50 | 3 | 2.12 | + | 0.17 | 0.02 | 129 | 318 | 6.35 | 4.63 |
| 20 | Woman | 42 | 3 | 0.32 | - | 0.4 | 0.04 | 127 | 216 | 4.42 | 4.21 |
| 21 | Woman | 6 | 3 | 1.32 | + | 0.58 | 0.11 | 115 | 91 | 9.03 | 4.15 |
| 22 | Woman | 23 | 3 | 0.43 | - | 0.73 | 0.65 | 120 | 222 | 3.49 | 4.72 |
| 23 | Woman | 31 | 3 | 5.42 | + | 1.21 | 0.98 | 121 | 290 | 9.6 | 5.94 |
| 24 | Man | 41 | 3 | 0.21 | - | 0.23 | 0.09 | 108 | 324 | 8.73 | 3.95 |
| 25 | Man | 43 | 4 | 8.86 | + | 0.28 | 0.02 | 125 | 210 | 4 | 3.96 |
| 26 | Woman | 23 | 4 | 36.25 | + | 0.61 | 0.11 | 129 | 139 | 4.86 | 4.72 |
| 27 | Woman | 29 | 4 | 53.13 | + | 0.69 | 0.12 | 133 | 164 | 3.65 | 4.45 |
| 28 | Woman | 33 | 4 | 2.31 | + | 0.61 | 0.11 | 136 | 195 | 7.03 | 4.65 |
| 29 | Woman | 63 | 4 | 0.28 | + | 0.86 | 0.2 | 112 | 286 | 7.81 | 3.97 |
| 30 | Woman | 31 | 4 | 3.6 | + | 0.32 | 0.03 | 106 | 118 | 3.77 | 2.75 |
| 31 | Woman | 24 | 4 | 23.91 | + | 0.65 | 0.2 | 115 | 196 | 2.43 | 4.76 |
| 32 | Woman | 21 | 4 | 10.07 | + | 0.64 | 0.11 | 105 | 353 | 3.71 | 5.4 |
| 33 | Woman | 13 | 4 | 0.08 | + | 0.86 | 0.13 | 133 | 395 | 5.26 | 4.47 |
| 34 | Woman | 37 | 4 | 4.38 | + | 0.44 | 0.16 | 127 | 216 | 4.42 | 4.21 |
| 35 | Woman | 43 | 4 | 3.15 | + | 0.48 | 0.18 | 121 | 294 | 4.43 | 4.37 |
|  |  |  |  |  |  |  |  |  |  |  |  |
|  |  |  |  |  |  |  |  |  |  |  |  |
| **Active patients** | | | | | | | | | | | |
| **No** | **Sex** | **Age** | **SLEDAI** | **Anti-dsDNA** | **ANA** | **C3** | **C4** | **Hb** | **PLT** | **WBC** | **RBC** |
| 1 | Woman | 38 | 5 | 1.33 | + | 0.81 | 0.12 | 144 | 33 | 6.7 | 4.89 |
| 2 | Woman | 31 | 5 | 2.53 | + | 0.14 | 0.17 | 108 | 184 | 3.28 | 4.25 |
| 3 | Man | 26 | 5 | 0.2 | + | 0.61 | 0.08 | 79 | 424 | 7.23 | 5.15 |
| 4 | Woman | 21 | 5 | 2 | + | 0.21 | 0.02 | 113 | 305 | 5.19 | 4.46 |
| 5 | Woman | 36 | 6 | 15.37 | + | 0.37 | 0.02 | 101 | 236 | 2.83 | 3.91 |
| 6 | Woman | 39 | 6 | 0.18 | + | 0.6 | 0.1 | 112 | 261 | 5.81 | 3.74 |
| 7 | Woman | 39 | 6 | 1.8 | + | 0.59 | 0.16 | 112 | 261 | 5.81 | 3.74 |
| 8 | Woman | 37 | 6 | 115.71 | + | 0.26 | 0.06 | 106 | 121 | 4.34 | 3.72 |
| 9 | Woman | 64 | 6 | 2.98 | + | 0.58 | 0.14 | 101 | 393 | 19.39 | 4.89 |
| 10 | Woman | 34 | 6 | 0.15 | + | 0.68 | 0.22 | 109 | 255 | 9.48 | 4.17 |
| 11 | Woman | 70 | 6 | 11.27 | + | 0.52 | 0.2 | 67 | 324 | 9.01 | 2.57 |
| 12 | Woman | 29 | 7 | 13.78 | + | 0.29 | 0.04 | 131 | 142 | 3.85 | 4.58 |
| 13 | Woman | 63 | 7 | 0.1 | - | 0.95 | 0.18 | 133 | 215 | 5.1 | 5.19 |
| 14 | Woman | 46 | 8 | 0.28 | + | 0.52 | 0.15 | 134 | 247 | 14.64 | 4.8 |
| 15 | Woman | 16 | 8 | 95.02 | + | 0.46 | 0.08 | 104 | 322 | 4.98 | 4.08 |
| 16 | Woman | 57 | 8 | 3.74 | + | 0.41 | 0.03 | 117 | 160 | 4.93 | 4.09 |
| 17 | Woman | 46 | 8 | 1.17 | + | 0.65 | 0.17 | 113 | 217 | 5.88 | 3.94 |
| 18 | Woman | 63 | 8 | 80 | + | 0.46 | 0.05 | 85 | 190 | 3.63 | 3.24 |
| 19 | Woman | 59 | 8 | 16.47 | + | 1.28 | 0.32 | 112 | 286 | 7.81 | 3.97 |
| 20 | Woman | 32 | 8 | 40.38 | + | 0.44 | 0.16 | 54 | 43 | 4.22 | 1.85 |
| 21 | Woman | 23 | 8 | 1.25 | + | 0.53 | 0.07 | 79 | 89 | 2.39 | 2.65 |
| 22 | Woman | 22 | 9 | 35.14 | + | 0.57 | 0.1 | 101 | 371 | 3.94 | 3.89 |
| 23 | Woman | 25 | 9 | 3.99 | + | 0.51 | 0.11 | 122 | 329 | 10.61 | 4.5 |
| 24 | Woman | 36 | 10 | 2.2 | + | 0.35 | 0.08 | 93 | 428 | 6.35 | 3.46 |
| 25 | Woman | 15 | 10 | 10.95 | + | 0.33 | 0.05 | 76 | 196 | 8.98 | 2.53 |
| 26 | Woman | 45 | 10 | 24.64 | + | 0.77 | 0.1 | 114 | 258 | 19.03 | 3.92 |
| 27 | Woman | 26 | 10 | 7.17 | + | 0.24 | 0.07 | 105 | 152 | 5.25 | 3.88 |
| 28 | Woman | 24 | 10 | 3.5 | + | 0.42 | 0.11 | 115 | 253 | 3.01 | 4.02 |
| 29 | Woman | 30 | 10 | 15.38 | + | 0.43 | 0.04 | 115 | 294 | 5.35 | 3.65 |
| 30 | Man | 34 | 11 | 1.57 | + | 0.41 | 0.05 | 137 | 159 | 2.78 | 4.97 |
| 31 | Woman | 26 | 11 | 70.84 | + | 0.35 | 0.02 | 117 | 8 | 3.95 | 3.93 |
| 32 | Woman | 27 | 12 | 10.22 | + | 0.34 | 0.04 | 124 | 197 | 3.57 | 4.07 |
| 33 | Woman | 17 | 12 | 14.74 | + | 0.09 | 0.02 | 83 | 156 | 4.42 | 2.82 |
| 34 | Woman | 18 | 12 | 78.42 | + | 0.48 | 0.18 | 83 | 4 | 5.81 | 3.02 |
| 35 | Woman | 32 | 13 | 19.84 | + | 0.44 | 0.05 | 109 | 132 | 2.32 | 3.83 |
| 36 | Woman | 31 | 14 | 7.7 | + | 0.34 | 0.06 | 79 | 234 | 9.72 | 2.65 |
| 37 | Woman | 33 | 15 | 37.72 | + | 0.15 | 0.04 | 69 | 87 | 1.85 | 3.88 |
| 38 | Woman | 21 | 15 | 6.6 | + | 0.27 | 0.05 | 67 | 60 | 3.46 | 2.82 |
| 39 | Woman | 42 | 16 | 31 | + | 0.46 | 0.11 | 120 | 124 | 11.46 | 3.49 |
| 40 | Man | 23 | 16 | 10 | + | 0.25 | 0.06 | 108 | 307 | 5.33 | 3.75 |
| 41 | Woman | 48 | 16 | 33.12 | + | 0.13 | 0.02 | 102 | 22 | 1.94 | 3.34 |
| 42 | Woman | 22 | 18 | 18.72 | + | 0.66 | 0.08 | 97 | 203 | 6.61 | 3.24 |
| 43 | Woman | 36 | 19 | 2.06 | + | 0.38 | 0.07 | 110 | 236 | 2.83 | 3.91 |
| 44 | Man | 7 | 19 | 43.48 | + | 0.21 | 0.03 | 106 | 269 | 4.27 | 4.15 |
| 45 | Woman | 65 | 19 | 26.85 | + | 0.52 | 0.09 | 80 | 352 | 3.65 | 2.85 |
| 46 | Woman | 35 | 19 | 6.44 | + | 0.33 | 0.03 | 103 | 160 | 3.32 | 3.13 |
